# The In Vitro and Vivo Validation of a New Ultrasound Method to Quantify Thoracolumbar Fascia Deformation

**DOI:** 10.3390/jcm14051736

**Published:** 2025-03-04

**Authors:** Andreas Brandl, Robert Schleip

**Affiliations:** 1 Conservative and Rehabilitative Orthopedics, TUM School of Medicine and Health, Technical University of Munich, 80333 Munich, Germany; robert.schleip@tum.de; 2 School of Osteopathy, College Sutherland, 22769 Hamburg, Germany; 3 Department for Medical Professions, Diploma Hochschule, 37242 Bad Sooden-Allendorf, Germany

**Keywords:** thoracolumbar fascia, deformation, ultrasound, validity, acute low back pain

## Abstract

**Background:** A new method for quantifying thoracolumbar fascia deformation (TLFD) and its shear capacity has been introduced, and its reliability for discriminating patients with low back pain (LBP) from healthy controls has been demonstrated in a recent paper. The aim of this study was to investigate the method in terms of criterion validity. **Methods:** First, the concurrent validity of the TLFD ultrasound measurement method (TLFD_US) was tested in vitro, using a custom-made tissue sliding device that mimics tissue shearing and generates ground truth data. Second, ultrasound images and videos of TLFD were acquired from 10 acute LBP patients and 10 healthy controls by a blinded assessor. In vivo, the concurrent validity of TLFD_US and speckle tracking analysis was then tested. Third, the contribution of the surrounding tissue layers of the erector spinae muscle and dermis to TLFD was calculated using multiple linear regression. **Results:** The in vitro concurrent validity between TLFD_US and ground truth was excellent (ICC = 0.99; *p* < 0.001). In vivo, the concurrent validity between TLFD_US and speckle tracking analysis was large (r = 0.701; *p* < 0.001). Multiple linear regression revealed a large effect regarding the relationship between dermis shear and TLFD (R^2^ = 0.353; *p* = 0.01). **Conclusions:** TLFD_US showed excellent criterion validity. Its suitability for capturing morphological parameters of the thoracolumbar fascia is further reinforced.

## 1. Introduction

One of the leading causes of disability-related loss of life years worldwide is low back pain (LBP). It therefore represents a significant global economic burden on healthcare systems [1]. While LBP-related pain syndromes are estimated to cost USD 135 billion in the United States [2], in Germany, where the study was conducted, they are the most common condition, with a 14% share of disability-related loss of life years [3]. In addition, the cure rate for acute LBP (aLBP) is only one third. The remainder, i.e., the majority of all aLBP patients, suffer new episodes of pain within the next year [4].

Besides some risk factors such as older age, higher BMI and greater physical exertion or in particular previous phases of aLBP [5], there is increasing evidence that the thoracolumbar fascia (TLF) may play a contributory role in the development of low back pain [6,7,8,9]. The TLF is an aponeurotic, diamond-shaped structure that covers the paraspinal muscles and separates them from the abdominal muscles with a layer. The most posterior layer is dominated by the insertion of the latissimus dorsi muscle [10]. Its rich innervation, involving in particular type A and C nociceptive nerve fibers, makes it susceptible to pathophysiological changes [8,11]. In recent years, a number of potential causes of nociceptor sensitization have been identified: the loss of elasticity and lubricity [6], micro-injuries [12], swelling due to overuse [13], and even indications that psychosocial factors may considerably reduce the fascial ability to shear or its stiffness [14,15,16].

In view of these TLF-LBP relationships, ultrasound (US) methods have been developed to quantify the sliding and deformation properties of the TLF [6,17,18,19,20]. Two generally different approaches have been developed for these methods. One is the use of US measurements for landmarks of prominent anatomical structures [19,21], and the other is the use of automated methods based on speckle tracking and cross-correlation functions [6,17]. We have recently introduced a new, landmark-based easy-to-use method for diagnosis and treatment control in daily practice. Its intra- and inter-rater reliability in quantifying TLF deformation (TLFD) was therefore demonstrated in a comprehensive study [21]. For a detailed theoretical framework of the method, its intra- and inter-rater reliability, and its clinical relevance, please see Brandl et al. [21]. However, to date, it is neither clear that the absolute distance value of the TLFD US measurement (TLFD_US) corresponds to the real distance nor in which tissue layers (dermis and erector spinae muscle) the deformation occurs and in what proportion.

This work follows on from our previous reliability study, in which we described in more detail the rationale for a new ultrasound measurement method for assessing TLF properties and outlined its relevance for clinical use and further practical application. The aim of the present study was to investigate the criterion validity of the TLFD_US method in terms of a comparison between the measurements within the ultrasound images and real-world data. Therefore, the concurrent validity of the ground truth data in vitro and the speckle tracking analysis in vivo should be evaluated. In addition, the sliding of the TLF in other tissue layers should be determined as a secondary objective with the speckle tracking analysis that defines the TLFD.

## 2. Materials and Methods

The work presented here was a criterion validity study and part of a larger project investigating the neuromotor associations of the TLF. The project was prospectively registered with the German Registry for Clinical Trials (DRKS00027074) and was reviewed and approved for in vivo validation with human participants by the ethics committee of the Diploma University of Applied Science (No. 1014/2021). This study was conducted in accordance with the Declaration of Helsinki and written informed consent was obtained from the participants.

### 2.1. Ultrasound Measurement of the Thoracolumbar Fascia Deformation

The TLFD_US using the junction between the latissimus dorsi muscle and the TLF as an anatomical landmark has already been described by Brandl et al. [21,22,23]. A detailed description of the ultrasound examination protocol can be found at https://dx.doi.org/10.17504/protocols.io.eq2lyjbmwlx9/v1, accessed on 4 February 2025.

**Figure 1:**
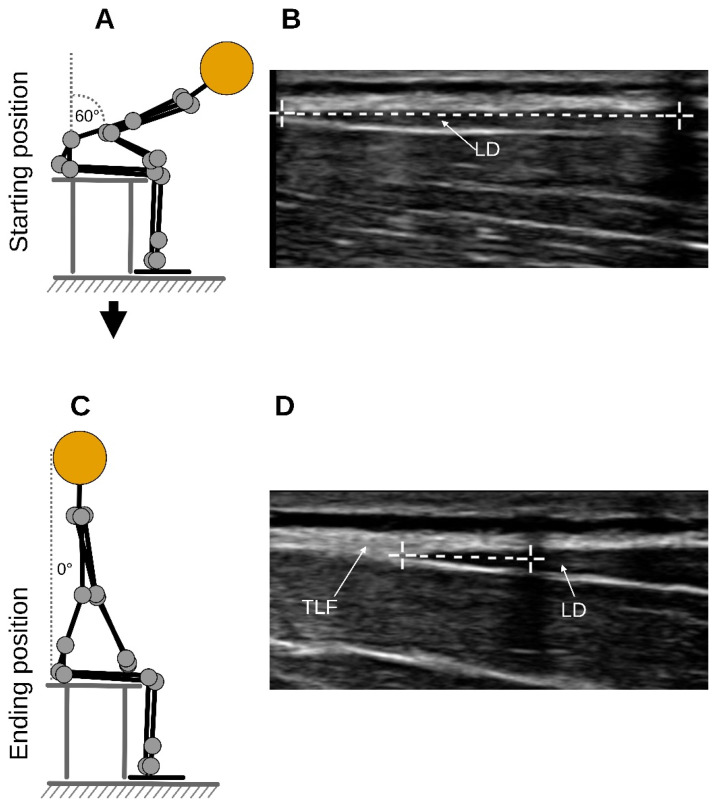
The measurement procedure. (**A**) The flexion phase of the trunk. (**B**) The measurement time point at the starting position. (**C**) The fully extended position of the trunk extension task. (**D**) The measurement time point at the ending position. The left white cross on the measurement line marks the LD/TLF junction. The white cross on the right marks the center of the artificial reference created by reflective tape on the skin. TLF, thoracolumbar fascia; LD, latissimus dorsi muscle.

Briefly, each participant sat on a treatment table and flexed their trunk to an approximate flexion angle of 60 degrees (starting position) as determined by the examiner, and here, a static US image was acquired (Clarius L15 HD3, 5–15 MHz Linear Transducer, Vancouver, BC, Canada; Figure 1A,B). Participants then extended their trunk to 0 degrees, which was the ending position of the trunk extension task (TET), and a static, secondary US image was acquired again (Figure 1C,D). The measurement was the distance between the junction of the latissimus dorsi muscle with the TLF and an artificial reference created by reflective tape on the skin shown in the images (Figure 1B,D). The difference between the distances of the starting and the ending position represents the deformation of the TLF (Figure 1B,D).

In a comprehensive recent reliability study by our research group, intra-rater reliability was rated as excellent, with an ICC of 0.92 and a minimal detectable change of 5.54 mm (*p* < 0.001). The inter-rater reliability was slightly worse, with a good ICC of 0.78 and a minimal detectable change of 8.70 mm (*p* < 0.001). Furthermore, a cut-off point of 6 mm was determined to differentiate LBP patients from healthy individuals, meaning that a TLFD of less than 6 mm identified 100% of LBP patients (sensitivity) and more than 6 mm identified 93.75% of healthy individuals (specificity) [21].

### 2.2. In Vitro Concurrent Validity with Ground Truth

**Figure 2:**
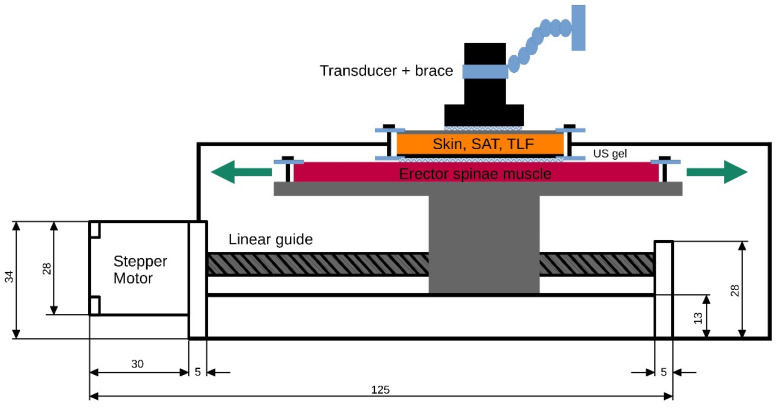
The tissue sliding device. The device consists of a computer-controlled linear drive with a ball screw on which the imitation erector spinae gel pad is connected to the overlying layers in a freely movable manner. The ultrasound transducer, which is aligned at right angles using a spirit level, is permanently installed with a holder. The transducer and each gel pad are separated by ultrasound gel. A detailed device description with additional video material and the generation of ground truth data is publicly available in the Zenodo repository: https://zenodo.org/records/11402043, accessed on 4 February 2025.

To generate ground truth data for the subsequent validation of the TLFD_US, a custom-built tissue sliding device was constructed. A polyurethane gel pad that mimics the erector spinae muscle is attached to a plateau mounted on a linear actuator (SFU1605-100 mm, Shandong Sair Mechanical Guide Co., Ltd., Gaotang, China) and driven by a high-precision stepper motor (NEMA17, ACT Motor GmbH, Bremen, Germany). This configuration allows the gel pad to move laterally under another gel pad that mimics the skin and SAT (Figure 2).

The gel pads (21 cm × 31 cm; Technogel GmbH, Berlingrode, Germany), which represent a two-layer phantom model of the lumbar tissues with its typical thickness and stiffness, were manufactured on the basis of properties described in the literature. For a detailed description of the development, properties and application, see Bartsch et al. [24] and Brandl et al. [25].

Between the gel pads, ultrasound gel (DocCheck Ultrasound Gel, Gello GmbH Geltechnik, Ahaus-Wüllen, Germany) for reducing friction and enabling smooth gliding was attached. At each gel pad, a strain gauge sensor (ZD10-100, Shenzhen Lanqi Technology Co., Ltd., Shenzhen, China) was attached to measure any shear deformation during their relative movement. Equipped with a digital caliper, the stepper motor maintained constant speeds with a positioning accuracy of ± 1 µm. This ensured the reliable documentation of the displacement of each gel pad relative to the other. No shear deformation was detected within the gel pads when determining the ground truth data (<0.1 mm). The measurement uncertainty for the absolute distance between the gel pads was ±2 µm, k = 2 (95% confidence interval), in accordance with the “Guide to the expression of uncertainty in measurement” [26].

With this setting, 36 distances between 3 and 20 mm with a constant shear rate of 3.15 mm/s were recorded. In addition, 10 different velocities from 2.41 mm/s to 5.36 mm/s were recorded for a distance of 20 mm. These distances and speeds correspond to the typical TLFD and trunk extension velocities of 200 LBP patients and healthy individuals that we previously assessed [27]. The complete ground truth dataset and epidemiologic data as well as a detailed description of the device including the design description are available at https://zenodo.org/records/11402043, accessed on 4 February 2025.

**Figure 3:**
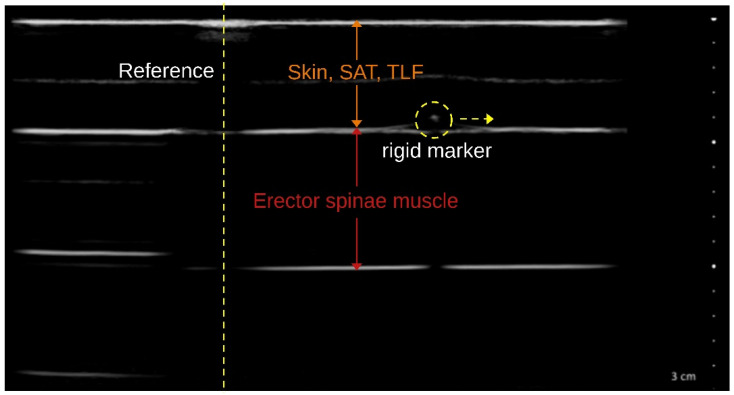
Two-layer phantom tissue ultrasound imaging. Skin, SAT, TLF: The first gel pad, which was attached to the tissue sliding device, represents the cutis, the subcutaneous adipose tissue and the thoracolumbar fascia. The second gel pad represents the erector spinae muscle with its marker displaced laterally by the linear actuator (yellow dashed circle). The other marker served as a reference (yellow dashed line). A detailed description of the imaging procedure with additional video material and the generation of ground truth data is publicly available in the Zenodo repository: https://zenodo.org/records/11402043, accessed on 4 February 2025.

The TLFD_US of the distances corresponding to each ground truth was performed with a reference marker in each phantom tissue layer clearly visible in the US image (Figure 3). Images were acquired simultaneously with the generation of ground truth data for all 36 distances and 10 velocities using the tissue sliding device with an attached US transducer by a blinded examiner with a total of 10 years of practice in US examinations. Subsequently, the measured distances were compared with the respective real value by an evaluator who was also blinded.

### 2.3. In Vivo Concurrent Validity with Speckle Tracking Analysis

To assess the concurrent validity of the manual TLFD_US and the automated speckle tracking analysis commonly used in research, 10 aLBP patients and 10 healthy individuals were studied. The reliability of speckle tracking methods for fascial shearing was previously tested by Langevin et al. (ICC = 0.98) [6] and Tomita et al. (ICC = 0.95) [17] for the TLF and by Ellis for the tibial nerve (ICC = 0.75) [28].

We calculated the sample size of 20 participants based on the least acceptable correlation to define concurrent validity between the methods of r = 0.60 [29], a significance level of 0.05, and a statistical power of 0.80 [30]. Inclusion criteria for the aLBP group according to the European guidelines for the treatment of aLBP (<6-week pain duration) were a visual analog scale score higher than 3 and an Oswestry disability score higher than 10. Healthy individuals had no pain episodes or physician visits for LBP in the last 5 years. Exclusion criteria were age under 18 or over 60 years, operations or scars in the area of the TLF, skin changes (e.g., urticaria or neurodermatitis), medications that affect blood circulation or act as muscle relaxants, and rheumatic diseases.

**Figure 4:**
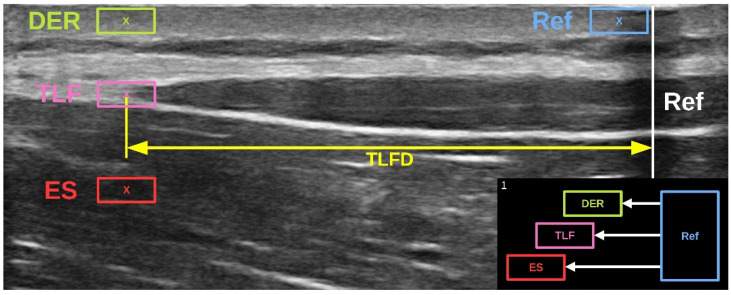
The speckle tracking analysis of lumbar tissue. The respective pixels of the regions of interest (4 × 7 mm rectangles) are tracked in an ultrasound video showing the trunk extension task. The displacement of DER, TLF, and ES from the Ref was post-analyzed using Kinovea software, version 0.9.5. Ref, artificial reference of reflective tape on the skin; DER, dermis; TLF, thoracolumbar fascia; ES, erector spinae muscle; TLFD, deformation of the TLF.

The investigator, who was blinded to the group membership, measured the TLFD sonographically from each participant as described in Section 2.1. A US video of the entire trunk extension task was then recorded from the TLF (for more details on this procedure, see Brandl et al. [23]). The videos were then tracked in post-analysis using Kinovea (version 0.9.5; Kinovea open source project, www.kinovea.org). The tracking pixels from the videos were tested for several scenarios, and Kinovea was recommended as a valid and reliable tool [31,32]. The individual speckle tracking of pixels was applied within a 4 × 7 mm rectangle representing the region of interest as described by Rodriguez et al. [33]. The analysis involved the determination of lateral tissue displacement from the starting to the ending position (Figure 1). The measured pixels were converted to millimeters using the scale bar in the US video and the Kinovea calibration function [31]. The procedure was performed simultaneously by the program for the different lumbar tissue layers (dermis, SAT, TLF, and erector spinae) and the artificial reference in the US video that we had previously defined (Figure 4).

### 2.4. Statistical Analysis

All data met the criteria for parametric testing. Descriptive statistics are reported as the mean, standard deviation (SD), minimum, maximum and 95% confidence interval (95% CI).

The intraclass correlation coefficient (ICC) between the in vitro TLFD_US and the distance ground truth was calculated using a 2-way random effects model (absolute agreement, ICC_(2,k)_, multiple raters, k = 2). The resulting values from the ICC calculation were categorized as “poor” (<0.50), “moderate” (0.50 to 0.75), “good” (0.75 to 0.90), and “excellent” (>0.90) according to Koo and Li [34]. A Bland–Altman plot was created to provide further visual information on the limits of agreement between the manual TLFD_US and the real values. The agreement between TLFD_US and the speed ground truth was presented in a Bland–Altman difference quantile–quantile plot (Q-Q) in addition to the descriptive evaluation.

The Pearson’s product moment correlation coefficient was calculated between in vivo TLFD_US and speckle tracking analysis and interpreted according to Cohen [35] as ”small” (0.1 to 0.3), “medium” (0.3 to 0.5) or “large” (0.5 to 1.0) correlations.

A multiple linear regression model with the speckle tracking analysis of TLF as the dependent variable and dermis as well as the erector spinae muscle sliding as independent variables was performed to predict TLFD adhesion properties to adjacent tissue layers. Effect sizes (adjusted R^2^) were interpreted according to Cohen as “small” (0.01–0.08), “medium” (0.09–0.24), and “large” (>0.25) [35].

The significance level was set at *p* = 0.05. Analyses were performed using Jamovi 2.3 (The jamovi project, https://www.jamovi.org, accessed on 4 February 2025).

## 3. Results

A total of 36 images for distance and 10 images for velocity were derived and analyzed to verify the concurrent validity of TLFD_US and ground truth in vitro. For in vivo validation, the concurrent validity of TLFD_US and speckle tracking analysis was performed in 20 participants.

### 3.1. In Vitro Concurrent Validity with Ground Truth

An excellent degree of agreement was found between TLFD_US and distance ground truth (ICC_(2,2)_ = 0.99, 95% CI [0.998, 0.999], F_(35.0, 36.0)_ = 1846, *p* < 0.001).

**Figure 5:**
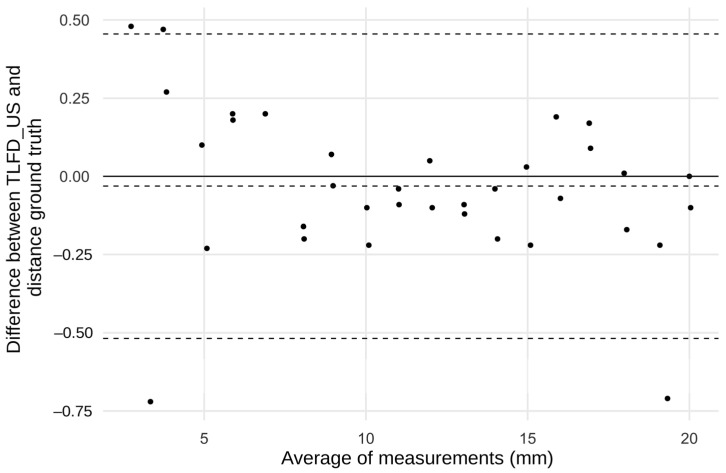
The Bland–Altman plot of the differences between TLFD_US and distance ground truth. TLFD_US, ultrasound measurement of the deformation of the thoracolumbar fascia.

The Bland–Altman diagram showed that almost all points were within the small limits of agreement. The mean difference was less than 0.05 mm, indicating that there were no systematic errors. Outliers exceeding the limits of agreement were only observed at the absolute end of the measurement range below 3.7 and above 19.6 mm (Figure 5).

**Figure 6:**
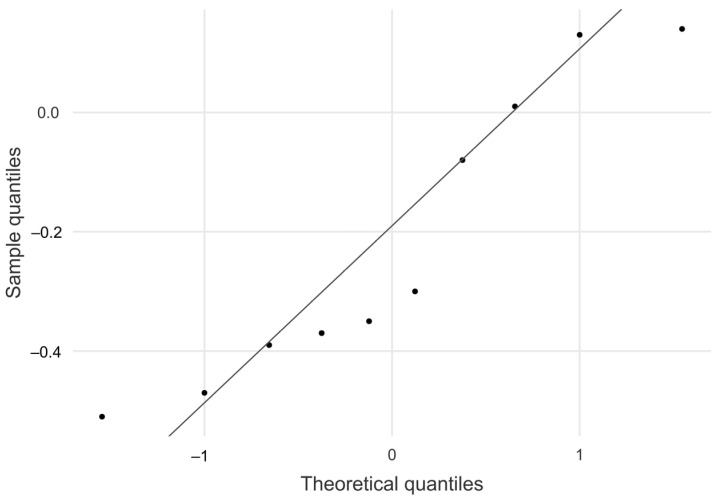
A quantile–quantile plot of the differences between TLFD_US and speed ground truth.

The absolute mean difference between TLFD_US and the speed ground truth was 0.215 mm, 95% CI [−0.410, −0.035]. As shown in the Q–Q plot, sample quantiles follow the theoretical quantiles and only deviate in a small range (Figure 6). Descriptive data for this analysis are shown in Table 1.

### 3.2. In Vivo Concurrent Validity with Speckle Tracking Analysis

A total of 20 participants were analyzed for the in vivo validation between TLFD_US and the speckle tracking analysis. The sample characteristics are listed in Table 2.

Pearson’s product moment correlation coefficient was large between the TLFD_US and speckle tracking analysis (r_(18)_ = 0.701; *p* < 0.001).

### 3.3. Thoracolumbar Fascia Deformation in Relation to Other Tissue Layers

The overall linear model fit was F_(2,17)_ = 6.19, *p* = 0.01, adjusted R^2^ = 0.353. The model explained 35% of the TLFD variability, emphasizing the significant influence of the included predictors and a large effect. Dermis sliding as a predictor of TLFD showed a significant effect (B = 0.780, 95% CI [0.15, 0.93]; standardized B = 0.541, 95% CI [0.22, 1.34]; *p* = 0.009). In contrast, erector spinae muscle sliding as a predictor of TLFD showed no significant effect (B = 0.386, 95% CI [−0.04, 0.81]; standardized B = 0.356, 95% CI [−0.03, 0.75]; *p* = 0.071).

## 4. Discussion

This is, to our knowledge, the first validation study to examine a US method for quantifying the sliding and deformation properties of the TLF. Given the growing evidence of a link between TLFD and LBP and the global economic burden of LBP, an in vitro validation was performed to determine whether the US measurements of TLFD correspond to real-world values. In addition, the method was validated in vivo in concurrence with speckle tracking analysis, which is commonly used in laboratory settings. A post-analysis of speckles from US videos was then used to deduce in which tissue layer surrounding the TLF (dermis and erector spinae muscle) did deformation occur and in what proportion.

### 4.1. In Vitro Concurrent Validity with Ground Truth

The degree of agreement between TLFD_US and the distance truth was excellent and, apart from a small area at the end of the measurement range, the US values matched the real values exactly. The TLFD_US is based on a trunk extension task performed by the participant. For ease of use in daily practice of this protocol, the speed at which the task is performed is only given in a rough range. Therefore, the accuracy of the method at different speeds was also investigated. The absolute mean difference was small and within a low 95% CI. To summarize, the TLFD_US has proven its high precision in an in vitro environment, and it can be assumed that the measured values represent the actual values.

Many authors have performed calculations of the physical properties of the TLF based on US analyses [6,17,18,19]. However, there is an urgent need to ensure that the calculations are based on valid data. This work therefore provides a trustworthy basis. In the course of developing the in vitro procedure, we have published the entire ground truth evaluation with the tissue sliding device in an open repository (https://zenodo.org/records/11402043, accessed on 4 February 2025). Work is currently underway with these data to validate certain speckle tracking analyses [27], and other authors are also invited to evaluate their various US methods of TLF against ground truth as well.

### 4.2. In Vivo Concurrent Validity with Speckle Tracking Analysis

TLFD_US and speckle tracking analysis showed large correlations. This analysis was performed in vivo with symptomatic aLBP patients and healthy individuals to reflect the population commonly seen in a physician’s office. Speckle tracking analysis using Kinovea, version 0.9.5, software in a recent study showed a high correlation of r = 0.97 with a minimal detectable change of 0.21 mm with the ground truth data, demonstrating similar concurrent in vitro validity [36]. The large in vivo relation of r = 0.70 between the two different methods proves the reliable use of the TLFD_US as a more practical and time-efficient measurement method for use in daily practice, which is well above the limit of the recommended minimum percentage agreement of 60% for diagnostic procedures in manual and musculoskeletal medicine [29,37]. In recent years, attempts have been made to characterize TLF properties using simple methods such as compressive stiffness (e.g., ultrasound elastography, myotonometry or intendometry) or TLF thickness measurements [38,39]. However, compressive stiffness is unlikely to detect stiffness changes in the thin TLF [24,25] and the measurement of TLF thickness is significantly influenced by the observer [40]. Furthermore, these measurements only consider one dimension in space, which gives TLFD_US an advantage as an additional method (a two-dimensional measurement of tissue sliding in an XY Cartesian coordinate system) for diagnosing and monitoring LBP [21].

### 4.3. Thoracolumbar Fascia Deformation in Relation to Other Tissue Layers

We performed multiple linear regression to determine which tissue layers the TLF deforms in vivo and to what extent. The overall model fit showed a large effect size in this regard, meaning that the included components of the dermis and erector spinae muscle significantly influenced TLFD. The linear model showed that mainly the dermis influenced TLFD. In total, 70% of the dermis sliding was transferred to the TLF. The 39% proportion of the erector spinae muscle was not significant in the modal but showed a trend with a *p*-value of 0.07.

Previous studies have primarily looked at the relationship between the TLF and the erector spinae muscle [6,23], just as most research on fascia has tended to focus on deeper layers of tissue [41]. In recent years, the role of the overlying layers of the TLF, particularly the superficial fascia, also known as Scarpa’s fascia, has been studied in more detail, and there is increasing evidence of the importance of these structures for the mechanical influence on the TLF [41,42]. Our study results are consistent with the observation of van Amstel et al. [43] on the direct effect of skin displacement on the range of motion of the spine, pelvis and hip. Wilke et al. [44] have demonstrated force transmission between muscle and superficial fascia. This study provides an initial indication that the tissue layers overlying the TLF may have a greater influence on TLFD and lumbar mobility than previously considered.

**Figure 7:**
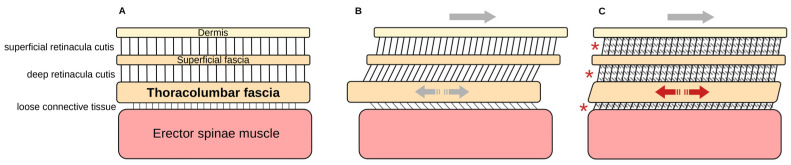
A model of thoracolumbar fascia deformation. (**A**) The extended position of the trunk. (**B**) Deformation during trunk flexion in healthy individuals. The dermis and the subcutaneous adipose tissue (represented as retinacula and superficial fascia) slide in relation to the TLF, which in turn is relatively fixed by the attached tissues, i.e., the latissimus dorsi muscle, the transversus abdominis muscle and the spinous processes. The erector spinae muscle can slide relatively freely under the TLF. In this respect, deformation is possible with the involvement of the surrounding connective tissue. (**C**) Deformation during trunk flexion in patients with low back pain. The red asterisks mark possible densification and/or the loss of liquid, which lead to adhesion zones with reduced gliding properties. The TLF cannot deform against the over- and underlying tissue layers. The internal shear stress on the TLF increases. TLF, thoracolumbar fascia.

Pirri et al. [19] and Willard et al. [10] have spoken of a kind of “frozen back” in LBP pathologies. Tomita et al. [17] calculated the internal shear strain of the TLF based on a Lagrangian approach to solid mechanics and found greater stress within the TLF in LBP patients. This sounds plausible considering that a decrease in sliding against the surrounding tissue must increase the shear strain within the TLF during movement (Figure 7). This could have inter-related pathological effects of various tissue overloads that lead to angiogenesis failure, hypoxia, inflammation, fibrosis, the excitation of nociceptors and others [9,45]. Our results of the influence of dermis on TLFD and, although not significant, a trend of the erector spinae muscle could confirm this hypothesis. However, due to the small sample size, no sub-analysis could be performed to separate the results of symptomatic and healthy participants. Further research could take this into account and use the effect sizes from our validity study in addition to the earlier study on intra- and inter-rater reliability to conduct more comprehensive follow-up work. It would be of particular interest to investigate the contribution of each tissue layer to TLFD, which should also include a heterogeneous group and appropriate subgroup analysis.

### 4.4. Limitations

This study has a number of limitations. First, various internal shear stresses occur during movement in vital biomaterials. This behavior can hardly be mimicked in vitro. We monitored the material deformation of the gel pad layers of the tissue sliding device and found no undesired shear stresses in the material. However, the approach is a two-dimensional simplification of real biomechanics in living subjects. Therefore, we also investigated its validity against the speckle tracking analysis in vitro with real patients and healthy individuals. The speckle tracking analysis showed strong validity against ground truth (r = 0.97) and small minimal detectable changes of 0.21 mm in a recent study [26]. This justifies the comparison of both methods in terms of their concurrent validity in this study. Third, we thoroughly calculated the sample size for the in vivo validation with a sufficient effect size to detect both statistically and clinically significant correlations. However, this calculation did not include a subgroup analysis to analyze aLBP patients and healthy individuals separately. This should be considered in future work.

## 5. Conclusions

TLFD_US showed excellent in vitro validity, and it can be assumed that the measurements also apply to the real-world data. Furthermore, it proved its resistance to variable tissue velocities with a stable measurement within small deviation ranges.

There was a large correlation between TLFD_US and speckle tracking analysis in an in vivo validation involving 10 aLBP patients and 10 healthy subjects. Additionally, speckle tracking analysis showed that TLFD was primarily caused by dermis sliding, although erector spinae muscle movement also appeared to have an impact. These results are a promising starting point for further research and may support the idea that LBP patients have a “frozen back”.

In addition to its previously established reliability, the TLFD_US has demonstrated its validity. When screening for LBP patients, the technique can be suggested to capture an extra morphologic TLF parameter.

## Figures and Tables

**Table 1:** Descriptive data of TLFD_US measurements and speed ground truth.

		95% CI				
	Mean	Lower	Upper	SD	Min	Max
Ground truth (mm)	20.0	20.0	20.0	0.006	20.0	20.0
TLFD_US (mm)	20.2	20.0	20.4	0.246	19.9	20.5

TLFD_US, ultrasound measurement of the deformation of the thoracolumbar fascia; SD, standard deviation; CI, confidence interval; Min, minimum; Max, maximum.

**Table 2:** Sample characteristics.

				95% CI		
	Group	N	Mean	Lower	Upper	SD
Sex (woman/men)	Healthy	10	6/4			
	aLBP	10	4–6 month			
Age (years)	Healthy	10	38.97	28.21	49.73	15.04
	aLBP	10	43.55	32.18	54.93	15.89
Weight (kg)	Healthy	10	66.04	54.92	77.16	15.55
	aLBP	10	75.52	66.31	84.73	12.87
Height (m)	Healthy	10	1.67	1.59	1.76	0.12
	aLBP	10	1.72	1.67	1.78	0.07
BMI	Healthy	10	23.48	20.29	26.67	4.45
	aLBP	10	25.57	22.35	28.79	4.50
VAS (mm)	aLBP	10	5.27	3.50	7.04	2.47
ODQ (0–100)	aLBP	10	49.40	37.35	61.45	16.84
Pain duration (days)	aLBP	10	9.10	6.44	11.76	3.72

aLBP, acute low back pain patients; Healthy, healthy individuals; BMI, body mass index; ODQ, Oswestry Disability Questionnaire.

## Data Availability

Data can be made available by the author upon request.

## Abbreviations

The following abbreviations are used in this manuscript:

aLBP	Acute low back pain
LBP	Low back pain
TLF	Thoracolumbar fascia
TLFD	Deformation of the thoracolumbar fascia
TLFD_US	Ultrasound method to measure the deformation of the thoracolumbar fascia
US	Ultrasound
